# Supplementary material for: Safe contact tracing for COVID-19: A method without privacy breach using functional encryption techniques based-on spatio-temporal trajectory data
Source: PLoS One. 2020 Dec 11;15(12):e0242758. doi: 10.1371/journal.pone.0242758 (PMC7732091; doi:10.1371/journal.pone.0242758)
Supplement: S1 Appendix — (PDF) [file pone.0242758.s002.pdf]

S1 Appendix. IPE encryption / decryption algorithm.

Algorithm 3: Setup

Input : Vector size n, prime number p,
two distinct cyclic groups of p order G_1, G_2

Output : master secret key msk

1 $g_1 \leftarrow$ random element in G_1
2 $g_2 \leftarrow$ random element in G_2
3 $B \leftarrow$ random $n * n$ matrix
4 $detB \leftarrow$ determinant of matrix B
5 $Bstar \leftarrow detB*$inverse matrix of B
6 $msk \leftarrow (detB, B, Bstar, g_1, g_2)$
7 return msk

Setup : set up the master secret key (msk)

Algorithm 4: Keygen

Input : Master secret key msk, plaintext $\vec{x}$, prime number p

Output : Secret key sk_x

1 $\vec{x} \leftarrow [\vec{x} \cdot \vec{x}, -2\vec{x}, 1]$
2 $detB, B, Bstar, g_1, g_2 \leftarrow msk$
3 $n \leftarrow len(\vec{x})$
4 $\alpha \leftarrow$ random value in the ring of integers modulo p
5 $k_1 \leftarrow [0, 0, ..., 0]$ k_1's length is n
6 **for** $j \leftarrow 0$ **to** $n-1$ **do**
7     $sum \leftarrow 0$
8   **for** $i \leftarrow 0$ **to** $n-1$ **do**
9         $sum \leftarrow sum + (\vec{x}[i] * B[i][j])$
10     $k_1[j] \leftarrow \alpha * sum$
11 **for** $i \leftarrow 0$ **to** $n-1$ **do**
12     $k_1[i] \leftarrow {g_1}^{k_1[i]}$
13 $k_2 \leftarrow {g_1}^{\alpha * detB}$
14 $sk_x \leftarrow (k_1, k_2)$
15 return sk_x

Keygen : generate secret key (sk_x)
6 - 10 : make $\alpha * \vec{x} \cdot B$
11 - 12 : make $k_1 = g_1^{\alpha * \vec{x} \cdot B}$
13 : make $k_2 = g_1^{\alpha * detB}$

Algorithm 5: Encryption

Input : Master secret key msk, plaintext $\vec{y}$, prime number p

Output : Ciphertext ct_y

1 $\vec{y} \leftarrow [1, \vec{y}, \vec{y} \cdot \vec{y}]$
2 $detB, B, Bstar, g_1, g_2 \leftarrow msk$
3 $n \leftarrow len(\vec{y})$
4 $\beta \leftarrow$ random value in the ring of integers modulo p
5 $c_1 \leftarrow [0, 0, ..., 0]$ c_1's length is n
6 **for** $j \leftarrow 0$ **to** $n - 1$ **do**
7     $sum \leftarrow 0$
8   **for** $i \leftarrow 0$ **to** $n - 1$ **do**
9         $sum \leftarrow sum + (\vec{y}[i] * Bstar[i][j])$
10     $c_1[j] \leftarrow \beta * sum$
11 **for** $i \leftarrow 0$ **to** $n - 1$ **do**
12     $c_1[j] \leftarrow {g_2}^{c_1[i]}$
13 $c_2 \leftarrow {g_2}^{\beta}$
14 $ct_y \leftarrow (c_1, c_2)$
15 return ct_y

Encryption : generate cipherTexts (ct_y)
6 - 10 : make $\beta * \vec{y} \cdot Bstar$
11 - 12 : make $c_1 = g_2^{\beta * \vec{y} \cdot Bstar}$
13 : make $c_2 = g_2^{\beta}$

Algorithm 6: Decryption

Input : A mapping function e satisfying condition C, secret key sk_x, ciphertext ct_y, maximum inner product value M

Output : dist(sk_x, ct_y)

1 $k_1, k_2 \leftarrow sk_x$
2 $c_1, c_2 \leftarrow ct_y$
3 $g \leftarrow e(k_1, c_1)$
4 $h \leftarrow e(k_2, c_2)$
5 $alpha \leftarrow ceiling(M^{0.5}) + 1$
6 $g_{inv} \leftarrow g^{-1}$
7 $t_b \leftarrow set$
8 **for** $i \leftarrow 0$ **to** $alpha$ **do**
9     $t_b[g^{i*alpha}] \leftarrow i$
10   **for** $j \leftarrow 0$ **to** $alpha$ **do**
11         $s \leftarrow h * {g_{inv}}^{j}$
12   **if** s **in** t_b **then**
13             $i \leftarrow t_b[s]$
14   return $(i * alpha + j)^{0.5}$
15 return -1

3: $g = e(g_1, g_2)^{\alpha\beta * B \cdot Bstar \cdot (\vec{x} \cdot \vec{y})} = e(g_1, g_2)^{\alpha\beta * detB * (\vec{x} \cdot \vec{y})}$

4: $h = e(g_1, g_2)^{\alpha\beta * detB}$

5-14: Baby-step giant-step algorithm for computing the discrete logarithm

Definition 2 ***Condition C*** *Given three distinct cyclic groups* G_1, G_2, G_T *of prime order* p*, let* $e : G_1 \times G_2 \rightarrow G_T$ *be a function that maps two elements from* G_1 *and* G_2 *onto a target group* G_T *with following properties*

1. *(Bilinear)* $\forall g_1 \in G_1, g_2 \in G_2, a, b \in \mathbb{Z}_p, e(g_1^a, g_2^b) = e(g_1, g_2)^{ab}$
2. *(Non-degenerate)* $e(g_1, g_2) \neq 1$
