# Supplementary material for: Safe contact tracing for COVID-19: A method without privacy breach using functional encryption techniques based-on spatio-temporal trajectory data
Source: PLoS One. 2020 Dec 11;15(12):e0242758. doi: 10.1371/journal.pone.0242758 (PMC7732091; doi:10.1371/journal.pone.0242758)
Supplement: Attachment — Submitted filename: letter.pdf [file pone.0242758.s004.pdf]

Answers to the Reviewers' Comments & Summary of Revision

Paper ID:
PONE-D-20-19705

Paper title:
Safe contact tracing for COVID-19: A method without privacy breach using functional encryption techniques based-on spatio-temporal trajectory data

Authors:
Wooil Kim, Hyubjin Lee, and Yon Dohn Chung

The authors would like to thank to the anonymous reviewers. In this document, we answer to the reviewers' comments and summarize the changes made in this revision.

Reviewer #1

Answers to the reviewer's comments

1) Alg 1 is the core contribution and its psuedocode and explanation can be substantially improved. (e.g., what exactly is meetFlag? or controls with breaks and continue's can be avoided, or differences between two append/add operations [step 15&31] can be shown)

- We appreciate the valuable comments of the reviewer. For better understanding, we revised the expressions of pseudocode and supplemented the explanation of the Alg 1 and Alg 2.

2) A better explanation for infection probability (Eq 1) can be provided.

- According to the reviewer's comment, we have added an explanation and a brief example of the infection probability (Eq 1) (Lines 140-145).

3) Provided that temporal domain (and the associated search) is 1-dimensional while spatial is 2, it is often more efficient to filter using time and refine with a spatial search. A clarification on why spatial search (location overlap) was performed first can be helpful.

- We use a location ID not the coordinate to check whether people have visited the same place or not. Thus both location and time are one-dimensional. While an equality check is necessary for the location, a distance computation is required on the time dimension. We clarify the location ID is used for a spatial search and why spatial search is performed first (Lines 145-147).

4) The time threshold (theta), can be instrumental in eliminating the resulting candidates. An explanation on how it can be used to speed up the search or further refine the test set can also be helpful. Alternatively, it can be shown via experiments that such parameter does or does not impact the results.<br>5) More experimentation with either real-life or synthetic datasets can be helpful as well in that what would be the impact of higher number of infections, how does the infection probability (or its parameters) impact the performance of the search?

- Thanks for the suggestion. We experimented with the impact of a higher number of infections and infection probability (by the time threshold) on the synthetic (Uniform distribution) dataset (Fig 6, Lines 212-223).

6) In Fig 1, line charts instead of area charts can be used, as it is not clear whether the charts are stacked or not. One could preferably use the log scale including the more current data.

- According to the comments, We switched the area chart to the line chart. Because of the scale problem, we deal with the daily new cases instead of total cases (Fig 1).

7) Pg. 2-Ln. 22-24. Steps can be rearranged to follow the progression (as in 3→4a→4b) or Fig 2 can be rearranged, as in Step 4a→3, Step 3→4a.

- According to the reviewer's comment, we rearranged the Fig 2 for a better understanding.

8) It is a bit confusing to use location as a pair of spatial point and time, since location is often used in purely spatial context.

- We revised the Def 1 and its explanation for a better understanding (Lines 104-107).

Reviewer #2

Answers to the reviewer's comments

1) On page 8, line32 please explain how the travel route data expose the privacy or what information the travel route data contains.

- Thanks for the valuable comments. According to the reviewer's comments, we supplemented an explanation and a brief example for a better understanding.

2) Please revise this sentence "No consideration of privacy even decreases the efficiency of epidemic controls because the suspected contacts might refuse to take infection tests because they are afraid of privacy breach". because is used frequently.

- We revised the sentence to "If privacy is not considered, people are reluctant to take COVID-19 infection tests, which makes the quarantine system less efficient" (Lines 41-43).

3) Please state what is a domain ?

- We clarify the expression in Table 2.

4) Line 181 , Line182 please provide specifications of the back-end server and version of python.

- We specified the version of python and django (Lines 189, 190).

5) Line191 The animation is available on the videos in Appendix. Please revise " The animation is available (in) the videos.

- We corrected the expression (Line 200).

6) Add another graph showing the cpu utilization comparison with varying time for the cores.

- As each core only computes the distance between ciphertext and secret key, the CPU-core utilization is monitored to be 100% during the processing. So, we supplemented the explanation for the CPU-core utilization instead of a graph (Lines 227, 228).

7) In references, Please write the accessed date i.e. when you are refereing to a website. e.g. Reference 20, 21 and 22 are without an accessed date.

- We added the accessed year to the website references (References 21-23).
