# Supplementary material for: Safe contact tracing for COVID-19: A method without privacy breach using functional encryption techniques based-on spatio-temporal trajectory data
Source: PLoS One. 2020 Dec 11;15(12):e0242758. doi: 10.1371/journal.pone.0242758 (PMC7732091; doi:10.1371/journal.pone.0242758)
Supplement: Attachment — Submitted filename: Plos one review.docx [file pone.0242758.s003.docx]

In this paper, the authors proposed a privacy-preserving contact tracing method based on spatio-temporal trajectory which can be practically used in many quarantine systems. In addition, a system is designed to visualize the contact tracing workflow. The paper has significant contributions amid the Covid-19 pandemic. I have a few Minor concerns given below:

1. On page 8, line#32 please explain how the travel route data expose the privacy or what information the travel route data contains.

2. Please revise this sentence "No consideration of privacy even decreases the efficiency of epidemic controls because the suspected contacts might refuse to take infection tests because they are afraid of privacy breach". because is used frequently

3. Please state what is a domain?

4. Line# 181, Line#182 please provide specifications of the back-end server and version of python.

5. Line#191 The animation is available on the videos in Appendix. Please revise " The animation is available (in) the videos.

6. Add another graph showing the CPU utilization comparison with varying time for the cores.

7. In references, please write the accessed date i.e. when you are referring to a website. e.g. Reference 20, 21 and 22 are without an accessed date.

Dr. Jehad ALI, Ajou University South Korea, Department of Computer Engineering.

Post-doctoral Fellow, Ajou University, South Korea.
